# Common bile duct polyp: an infrequent cause of jaundice and biliary obstruction

**DOI:** 10.1055/a-2271-5732

**Published:** 2024-03-08

**Authors:** Gerly E. Guzmán-Calderon, Carlos Huaraca, Brandon Bravo, Joseph Arzapalo

**Affiliations:** 1 279700 Gastroenterology, National Hospital Edgardo Rebagliati Martins, Lima, Peru; 2 538654 Gastroenterology, Clinica Anglo-Americana, Lima, Peru; 3 279700 Pathology, National Hospital Edgardo Rebagliati Martins, Lima, Peru

The most common etiology of common bile duct (CBD) obstruction is bile duct stones; less common causes include biliary polyps and intraductal papillary neoplasm of the bile duct (IPNB). Initially described by Nakamura et al. 1 in 2010, IPNB is defined as a pedunculated mass with intraluminal growth exhibiting significant malignant potential that can subsequently lead to cholangiocarcinoma.

We present the case of a 75-year-old woman with a history of jaundice and mild abdominal pain. Computed tomography and magnetic resonance cholangiopancreatography showed circumferential thickening of the proximal CBD and left hepatic duct ( Fig. 1 **,** Fig. 2 ). Liver function tests confirmed a cholestatic pattern, with total bilirubin of 7.4 mg/dL, direct bilirubin of 5.3 mg/dL, alkaline phosphatase of 475 IU/L, and normal CA 19.9 level. Cholangioscopy revealed a single whitish papillary mass with a regular surface, located between the proximal CBD and left hepatic duct, obstructing approximately 80% of the biliary lumen ( Video 1 ). Multiple samples were obtained using SpyBite forceps (Boston Scientific, Marlborough, Massachusetts, USA). Histological analysis confirmed the presence of an IPND with high grade dysplasia ( Fig. 3 **,** Fig. 4 ). The patient underwent left hepatectomy. The surgical specimen demonstrated a 17-mm lesion with biliopancreatic epithelium, involvement of the left hepatic duct, and no evidence of invasive carcinoma ( Fig. 4 ).

**Fig. 1:**
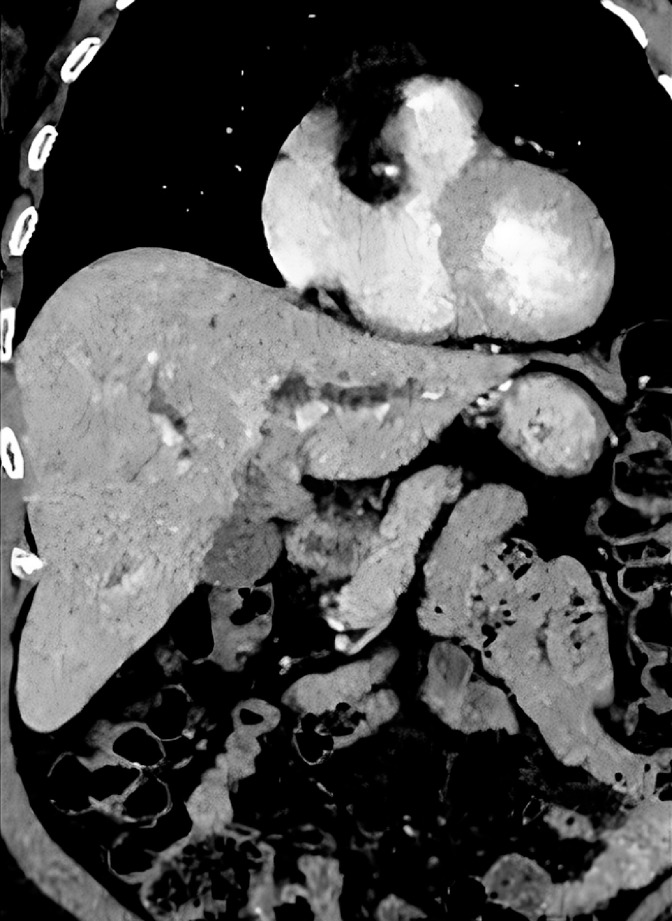
Computed tomography showing circumferential thickening of the proximal common bile duct and left hepatic duct.

**Fig. 2:**
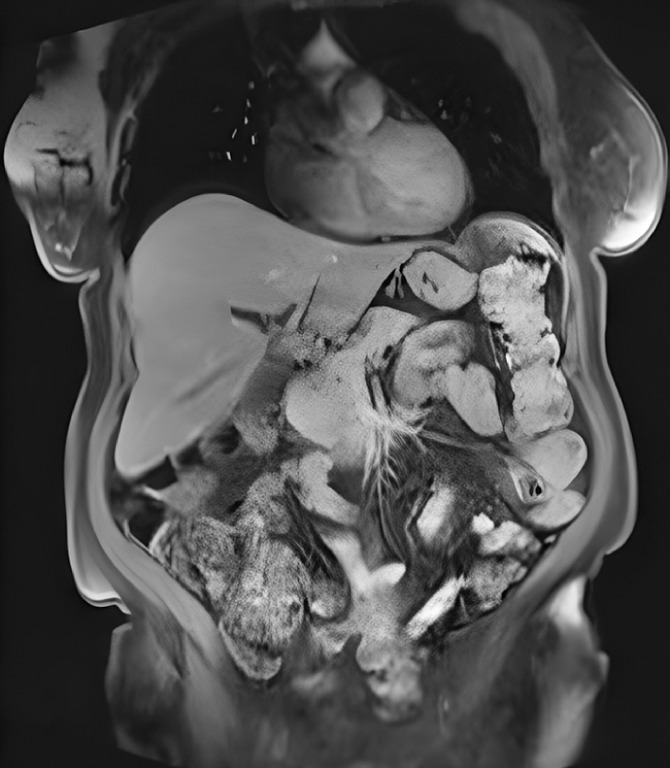
Magnetic resonance cholangiopancreatography showing circumferential thickening of the proximal common bile duct and left hepatic duct.

**Fig. 3:**
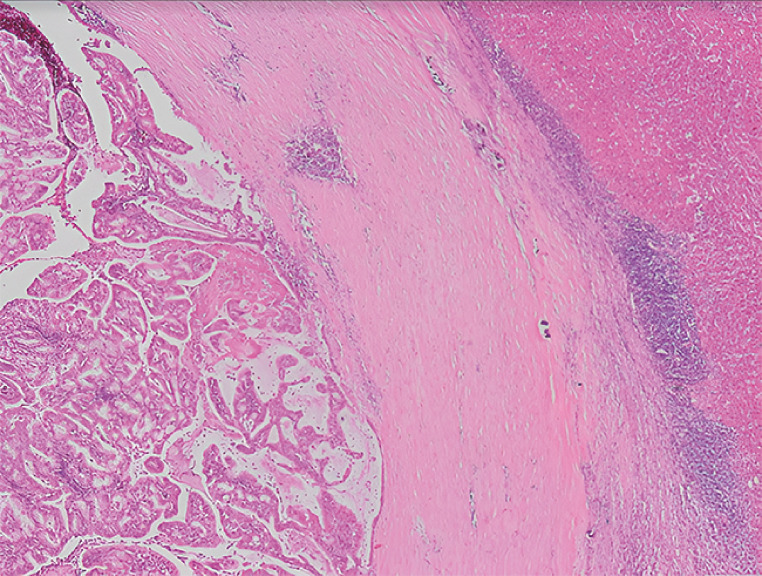
Histopathological image showing cubic monostratified papillary biliary epithelium and intraductal papillary neoplasia. Externally, a fibrous capsule delimits the liver tissue with an inflammatory infiltrate (hematoxylin and eosin, × 4).

**Fig. 4:**
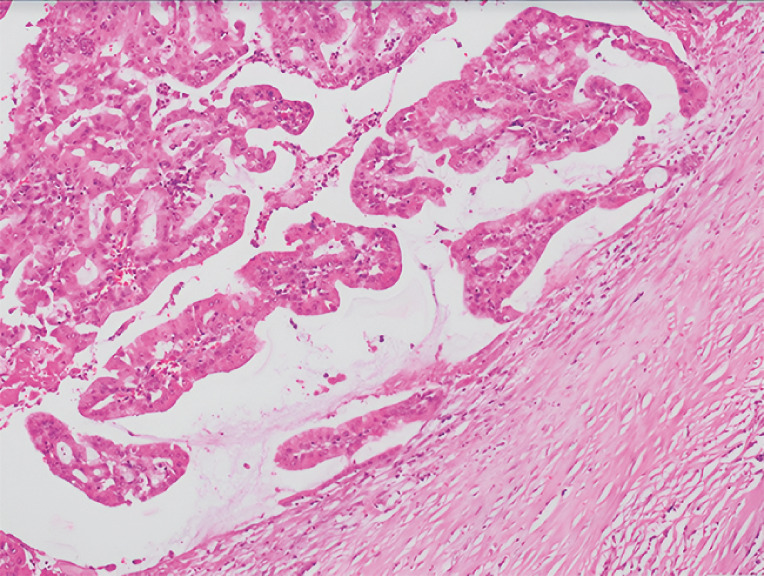
Histopathological image showing a papillary neoplasia with a fibrovascular core, as well as areas of high grade dysplasia in the gastric and pancreatobiliary epithelium, without evidence of invasive carcinoma (hematoxylin and eosin, 10×).

Cholangioscopy showing a single, whitish papillary mass with a regular surface, located between the proximal common bile duct (CBD) and left hepatic duct.Video 1

Biliary polyps are classified as IPNBs. Given their potential to cause obstructive jaundice and cholangitis, as well as a high malignant potential, IPNBs must be treated surgically 2 3 . Our case underscores the value of performing cholangioscopy with targeted biopsies for the assessment and characterization of CBD tumors.

Endoscopy_UCTN_Code_TTT_1AR_2AD

## Notes

Endoscopy E-Videos https://eref.thieme.de/e-videos
*E-Videos* is an open access online section of the journal *Endoscopy* , reporting on interesting cases and new techniques in gastroenterological endoscopy. All papers include a high-quality video and are published with a Creative Commons CC-BY license. Endoscopy E-Videos qualify for HINARI discounts and waivers and eligibility is automatically checked during the submission process. We grant 100% waivers to articles whose corresponding authors are based in Group A countries and 50% waivers to those who are based in Group B countries as classified by Research4Life (see: https://www.research4life.org/access/eligibility/ ). This section has its own submission website at https://mc.manuscriptcentral.com/e-videos .
